# Associations of DNA methylation estimators of protein abundance with concurrent and future physical health risk factors

**DOI:** 10.1038/s41598-025-31843-z

**Published:** 2025-12-10

**Authors:** Scott Waterfield, Paul Yousefi, Matthew Suderman

**Affiliations:** 1 MRC Integrative Epidemiology Unit, University of Bristol, Bristol, UK; 2 Population Health Sciences, Bristol Medical School, University of Bristol, Bristol, UK; 3 Cancer Research UK Integrative Cancer Epidemiology Programme, University of Bristol, Bristol, UK; 4 NIHR Bristol Biomedical Research Centre, University Hospitals Bristol and Weston NHS Foundation Trust and University of Bristol, Bristol, UK

**Keywords:** Epigenetics, DNA methylation, Proteomics, Physical health, Risk factors, Episcores, Longitudinal, ALSPAC, Predictive markers, Epidemiology, Risk factors, DNA methylation

## Abstract

DNA methylation (DNAm) is an epigenetic modification which plays a role in gene regulation and has genetic and environmental influences. Recently, DNAm-based models of protein abundance (termed episcores) have been developed and were found to be associated with incident disease in older adults. Here, we ask if these episcores are associated with latent physical health phenotypes in children and young adults in the Avon Longitudinal Study of Parents and Children (ALSPAC) cohort. Episcores were projected in ALSPAC participants who had DNAm measurements in cord blood, and peripheral blood at ages seven, nine, 17, and 24 (*n* = 192–2857). We analysed cross-sectional associations between 108 episcores and 17 physical health phenotypes, followed by an examination of prospective associations between episcores and the same phenotypes measured 2 + years after the blood samples used for episcore calculation. Two-sample Mendelian randomisation (2SMR) was then used to evaluate evidence for causal relationships between the underlying proteins and any associated physical health phenotypes. Of the associations tested between 17 physical health phenotypes and 108 episcores at multiple timepoints, 9 cross-sectional (CHIT1 is associated with 8 of these) and 11 prospective (SEMA3E is associated with 7 of these) phenotype-episcore associations were discovered. Of these, no 2SMR analyses suggested a causal effect of a protein on its related phenotype. We find evidence to suggest that episcores may be useful for discovering protein-phenotype associations in populations lacking direct measurements of protein abundance.

**Supplementary Information:**

The online version contains supplementary material available at 10.1038/s41598-025-31843-z.

## Introduction

DNA methylation (DNAm) is a biological process in which methyl groups are added to nucleotide bases of DNA. The most common form DNAm in mammals is methylation of a cytosine residue followed by guanine nucleotide in a linear sequence along the 5’ to 3’ direction of the DNA (known as a CpG site)^1^. DNAm can alter gene activity, most notably when it occurs in gene promoters where methylation is normally associated with repression of gene activity^2^. Biologically programmed DNAm plays an important role in normal human development, whilst aberrant DNAm has been associated with the onset of numerous diseases^3^. DNAm at both the global and single CpG site level is also known to be associated with environmental exposures^4^, the most well-known example being smoking, including a particularly strong association at a CpG site in the *AHRR* gene^5^. As a result, DNAm has received a lot of interest for its capacity to reflect both environmental and developmental information across an individual’s life span.

Recently, Gadd et al. trained linear models termed ‘episcores’ using DNAm levels at multiple CpG sites to estimate the abundance of 953 proteins^6^. They found that 109 episcores explained at least 1% of protein variance in test data (threshold: R > 0.10, P < 0.05). Gadd and colleagues then explored associations between these episcores and risk of 12 different morbidities, finding a total of 137 episcore-disease risk associations. Strikingly, a number of these associations involved episcores that only explained a small proportion (~ 1%) of proteomic variance. These findings suggest that episcores may be useful surrogates for protein variation that are predictive of health outcomes.

The Gadd episcores were trained and evaluated in older populations spanning 59–73 years of age, and their ability to predict phenotypes was restricted to serious diseases such as diabetes and heart disease. Following on from this study, we evaluated how well these episcores estimate proteomic measures in children aged 9, young adults (age ~ 24 years), and middle-aged adults in the Avon Longitudinal Study of Parents and Children (ALSPAC). We found that episcores correlated more strongly with measured plasma protein abundance in adults than in children^7^, but it is not known if they are able to detect phenotypic variance in younger general populations, particularly disease precursors (and comorbidities) like body size and inflammation.

We aim to address these gaps of knowledge by expanding the range of physical health phenotypes that episcores have been examined in relationship to in a population with measurements between birth and early adulthood. In particular, we calculate Gadd episcores in child participants of ALSPAC using DNAm data generated at birth in cord blood and at ages seven, nine, 17, and 24 in peripheral blood as part of the Accessible resource for integrated epigenomics studies (ARIES)^8–11^. We examine the prospective and cross-sectional associations between episcores and ‘physical health’ phenotypes ranging from body composition to cardiometabolic health measures. We then interrogate potential causal pathways of observed associations using 2-sample Mendelian randomisation (2SMR)^12^.

## Methods

### Study participants

ALSPAC is a prospective birth cohort study in south west England^8–10^. Pregnant women resident in one of the three Bristol-based health districts with an expected delivery date between April 1, 1991 and December 31, 1992 were invited to participate. The study has been described elsewhere in detail and ethical approval for the study was obtained from the ALSPAC Ethics and Law Committee and the Local Research Ethics Committees. ALSPAC initially enrolled a cohort of 14,451 pregnancies, from which 13,867 live births occurred in 13,761 women. Follow-up has included parent and child completed questionnaires, links to routine data and clinic attendance. The present analyses include first-born offspring participants only.

Research clinics were held when these offspring participants were approximately seven, nine, 10, 11, 13, 15, 18, and 24 years old. Data for 24 years of age were collected and managed using REDCap^13^ electronic data capture tools hosted at the University of Bristol. REDCap (Research Electronic Data Capture) is a secure, web-based software platform designed to support data capture for research studies. The study website contains details of all the data that is available through a fully searchable data dictionary https://www.bristol.ac.uk/alspac/researchers/our-data/.

As part of the ARIES project (http://www.ariesepigenomics.org.uk*)*, a sub-sample of ALSPAC mother–child pairs had DNA methylation measured using the Infinium HumanMethylation450 BeadChip platform (450k array). Here, we use DNA methylation data generated from cord blood, venous blood samples at age seven (mean age = 7.5, standard deviation (SD) = 0.15), age nine (mean age = 9.8, SD = 0.27), between age 15 and 18 (mean age = 17.7, SD = 0.4) which we term age 17 from this point, and 24 years (mean age = 24.4, SD = 0.75), creating up to six measures per child. All DNA methylation wet-lab and preprocessing analyses were performed at the University of Bristol as part of the ARIES project and has been described in detail previously^11^.

All analyses within this manuscript were carried out in accordance with the guidelines of the ALSPAC Ethics and Law Committee and the local research ethics committees. Informed consent was gathered from all participants, and individuals who have retracted their consent are removed from all analytical datasets without question. Informed consent was obtained from parents and guardians for minors within the study, and upon adulthood are given the right to withdraw their consent participation at any time without giving reason. More details can be found at: https://www.bristol.ac.uk/alspac/participants/.

### DNA methylation processing

DNAm data was processed using the meffil package (https://github.com/perishky/meffil/), using the default processing pipeline, including the functional normalisation step including slide number as a random effect to reduce batch effects.

### Epigenetic Estimation of protein expression

Gadd et al. used matched measurements of DNAm (450k array) and protein abundance panels (Somascan/Olink) to build epigenetic models of protein abundance^6^. Models were trained using elastic net penalised regression models in the KORA study (SomaScan proteins) and Lothian Birth Cohort 1936 (Olink proteins), with the protein measures as outcomes and DNAm as explanatory variables. Models were successfully fitted for 953 proteins and were then tested in external (or holdout) datasets. A total of 109 ‘episcore’ models passed performance thresholds (*R* > 0.1 and *P* < 0.05).

We used the Gadd et al. models to project 108 episcores in the ARIES DNAm data measured in cord blood at birth and in peripheral blood at ages seven years, nine years, 17, and 24 years. One of the 109 episcores could not be calculated due to missing data in the ARIES dataset.

### Physical health outcomes

Phenotypes were selected from two broad categories known to have important, well-known roles as biomarkers of physical health: body composition and cardiometabolic health. Body composition measures included BMI (Body Mass/Height^2^ (kg/M^2^)) and height (cm) for which there are measures recorded from birth which are extracted from electronic health records/parent surveys, and measures from the ALSPAC research clinics from age seven to 24. Dual energy X-ray absorptiometry (DXA) scans for measures of total body fat mass (g), total body lean mass (g), and total body bone mass (g) were assessed at ages nine, 11, 13, 15, 17, and 24 years. The cardiometabolic measures we analyse were recorded at various ALSPAC research clinics between the age of seven and 24 and are as follows: high density lipoprotein cholesterol (HDLc), low density lipoprotein cholesterol (LDLc), triglycerides, systolic blood pressure (SBP), diastolic blood pressure (DBP), pulse rate, insulin, glucose, lactate, citrate, acetate, and c-reactive protein (*CRP*), descriptive statistics of each variable are available in Supplementary Table 2.

### Covariates

Model covariates included are sex and blood cell count estimates. Cell counts were estimated using the Houseman method^14^ and appropriate published cell count references:

- Cell counts for cord blood were estimated using the reference generated by Gervin et al.^15^ including cord blood DNA methylation profiles for nucleated red blood cells, granulocytes, natural killer, CD14+, CD4T, CD8T, and B cells.
- Cell counts for peripheral blood were estimated using the reference generated by Reinius et al.^16^ including peripheral blood DNA methylation profiles for neutrophils, eosinophils, monocytes, natural killer, CD4T, CD8T, and B cells.

### Statistical analysis

A single model was used to carry out cross-sectional analyses, and two models were used to assess the association between episcores calculated using DNAm at a specific age and phenotypes recorded at a later date. Due to the use of a large number of episcores we carry out the univariate phenotype associations using MLM-based omic association (MOA)^17^, which accounts for episcore level correlation structure. The first model used in cross-sectional and prospective analyses is defined as:

$$\:Phenotype@t\:\sim\:Episcore@t\:+\:Sex\:+\:Cell\:Counts\:(\mathrm{C}\mathrm{r}\mathrm{o}\mathrm{s}\mathrm{s}-\mathrm{S}\mathrm{e}\mathrm{c}\mathrm{t}\mathrm{i}\mathrm{o}\mathrm{n}\mathrm{a}\mathrm{l})$$

$$\:Phenotype@t{\prime\:}\:\sim\:Episcore@t\:+\:Sex\:+\:Cell\:Counts\:\left(\mathrm{P}\mathrm{r}\mathrm{o}\mathrm{s}\mathrm{p}\mathrm{e}\mathrm{c}\mathrm{t}\mathrm{i}\mathrm{v}\mathrm{e}\right)$$

The second (‘phenotype-adjusted’) model is defined similarly but includes the phenotype measured at the same time as the DNAm as a covariate to control for phenotype autocorrelation, and as such is only run as a prospective model.

$$\:Phenotype@t{\prime\:}\:\sim\:Episcore@t\:+Phenotype@t\:+\:Sex\:+\:Cell\:Counts\: {\rm (Phenotype-adjusted\: Prospective).}$$

 

In each prospective model, time point t’ is at least 2 years after time point t. P-values were adjusted for multiple tests within each time point by calculating a false-discovery rate (FDR) (e.g. Phenotype@24 ~ episcore@7: 108 episcore x 17 phenotypes = 1836 tests). Phenotype-episcore associations with FDR < 0.05 were analysed in the phenotype-adjusted model. To maximise sample sizes and comparability between different phenotypes, the following ages were used (when available) for each phenotype outcome: seven, 10, 13, 17, and 24.

We quantified within-subject correlation for repeated measures (venous samples only (ages 7–24)) using intraclass correlation coefficients (ICC), estimated from random-intercept linear mixed models. For each outcome, we first fit a model of the form:

$$\:{Y}_{ij}=\mu\:+{u}_{i}+{\epsilon\:}_{ij}$$

where $\:{Y}_{ij}$is the outcome for individual $\:i$ at time point $\:j$, $\:\mu\:\:$is the population mean, $\:{u}_{i}\sim\:N(0,{\sigma\:}_{u}^{2})$is the subject-specific random intercept, and $\:{\epsilon\:}_{ij}\sim\:N(0,{\sigma\:}_{e}^{2})$is the residual error. The ICC was calculated as $\:{\sigma\:}_{u}^{2}/({\sigma\:}_{u}^{2}+{\sigma\:}_{e}^{2})$, reflecting the proportion of total variance explained by stable differences between individuals.

In cases where this initial model produced a singular fit (i.e. the estimated between-subject variance collapsed to zero), we refit the model including age as a fixed effect:

$$\:{Y}_{ij}=\mu\:+\beta\:{\hspace{0.17em}}{\mathrm{Age}}_{ij}+{u}_{i}+{\epsilon\:}_{ij}$$

Including age accounts for systematic changes in the outcome over time (e.g. growth in height), ensuring that the ICC represents the consistency of relative ranking between individuals rather than being dominated by age-related trends.

### Two-sample Mendelian randomization

Mendelian Randomization (MR) analysis relies on three fundamental assumptions:

- Relevance: the genetic variants selected are strongly associated with the exposures they are instrumenting.
- Independence: the genetic variants used as instruments are not associated with any confounding factors.
- Exclusion Restrictions: the genetic variants influence the outcomes exclusively through the exposures and not through any alternative pathways.

Causal effect estimates of proteins on physical health risk factors were calculated using two sample Mendelian randomization (2SMR). The TwosampleMR R package was used in conjunction with the OpenGWAS^18,19^ database for 2SMR analyses. We used the Sun BB et al.^20^ resource of pQTLs to identify instruments for proteins of interest (in each analysis, all valid pQTLs were used, alongside a cis-acting only pQTL analysis; cis-acting pQTLs were considered to be within 1 MB of the gene body). Genetic outcome associations were obtained from OpenGWAS using the following outcome IDs: Acetate - met-d-Acetate, Citrate - met-d-Citrate, Lactate - met-d-Lactate, fasting glucose - ebi-a-GCST90002232, fasting insulin - ebi-a-GCST90002238, DBP - ieu-b-39, LDLc - ieu-b-110, HDLc - ieu-b109, Triglyceride - ieu-b-111. None of the outcome instruments were from the study used to define exposure instruments (INTERVAL^20^. All genetic associations were extracted using a significance threshold of < 5e-08, a clumping r2 of 0.001, and a clumping distance cutoff of 10,000 kb. Mendelian randomization sensitivity analyses evaluated directionality (Steiger), heterogeneity (MR-egger) and pleiotropy (MR-egger). This paper was written according to the STROBE-MR guidelines^21^.

## Results

Characteristics of the study participants included in all analyses are provided in Table 1. There was a total of 3026 participants enrolled in ARIES with the following numbers of individuals DNAm profiled at each age group: birth (905), age seven (969), age nine (361), age 17 (2857), and age 24 (822), with 51.9% of participant being female. We note that the mothers of ARIES participants typically have higher educational background, are less likely to have smoked during pregnancy and slightly older than mothers of non-ARIES ALSPAC participants, as previously described^11^. The Intraclass correlation for the phenotypes used in this study ranged from 0.05 (SBP) – 0.58 (HDLc) (see Supplementary Table 1).

**Table 1:** Characteristics of ARIES participants eligible for analysis.

Outcome	Mean Range	SE Range	Sample Range (*N*)
Acetate	0.042, 0.059	0.00031, 0.0019	361, 2855
BMI	16.2, 25.1	0.07, 0.37	194, 2657
Bone	2,110, 2,790	10.4, 33.7	192, 2614
CRP	1.14, 2.13	0.073, 0.22	361, 2857
Citrate	0.09, 0.16	0.00042, 0.0015	361, 2855
DBP	57.8, 67.7	0.147, 0.595	196, 2545
Fat	13,300, 23,700	196, 832	192, 2614
Glucose	3.92, 4.31	0.008, 0.03	361, 2855
HDL	1.26, 1.56	0.005, 0.021	361, 2857
Height	126, 173	0.173, 6.59	194, 2658
Insulin	7.81, 10.4	0.114, 0.407	361, 2857
LDL	2.06, 2.42	0.0113, 0.038	361, 2857
Lactate	0.911, 1.36	0.00832, 0.0331	361, 2855
Lean	38,000, 48,000	198, 671	192, 2614
Pulse	64, 82.9	0.21, 0.94	196, 2545
SBP	107, 125	0.22, 0.84	196, 2545
Triglycerides	0.83, 1.06	0.0067, 0.030	361, 2857

### CHIT1 is associated with numerous physical health phenotypes in adolescence

Overall, we carried out a large-scale analysis of cross-sectional and prospective associations between 108 episcores and 17 physical health risk factors (*N* = 192–2857) across multiple cross-sectional and prospective time points, comprising a total of 26,568 associations within the cross-sectional and prospective models, and a subset (15,984) of these in the phenotype-adjusted prospective model (Supplementary Table 3).

**Fig. 1:**
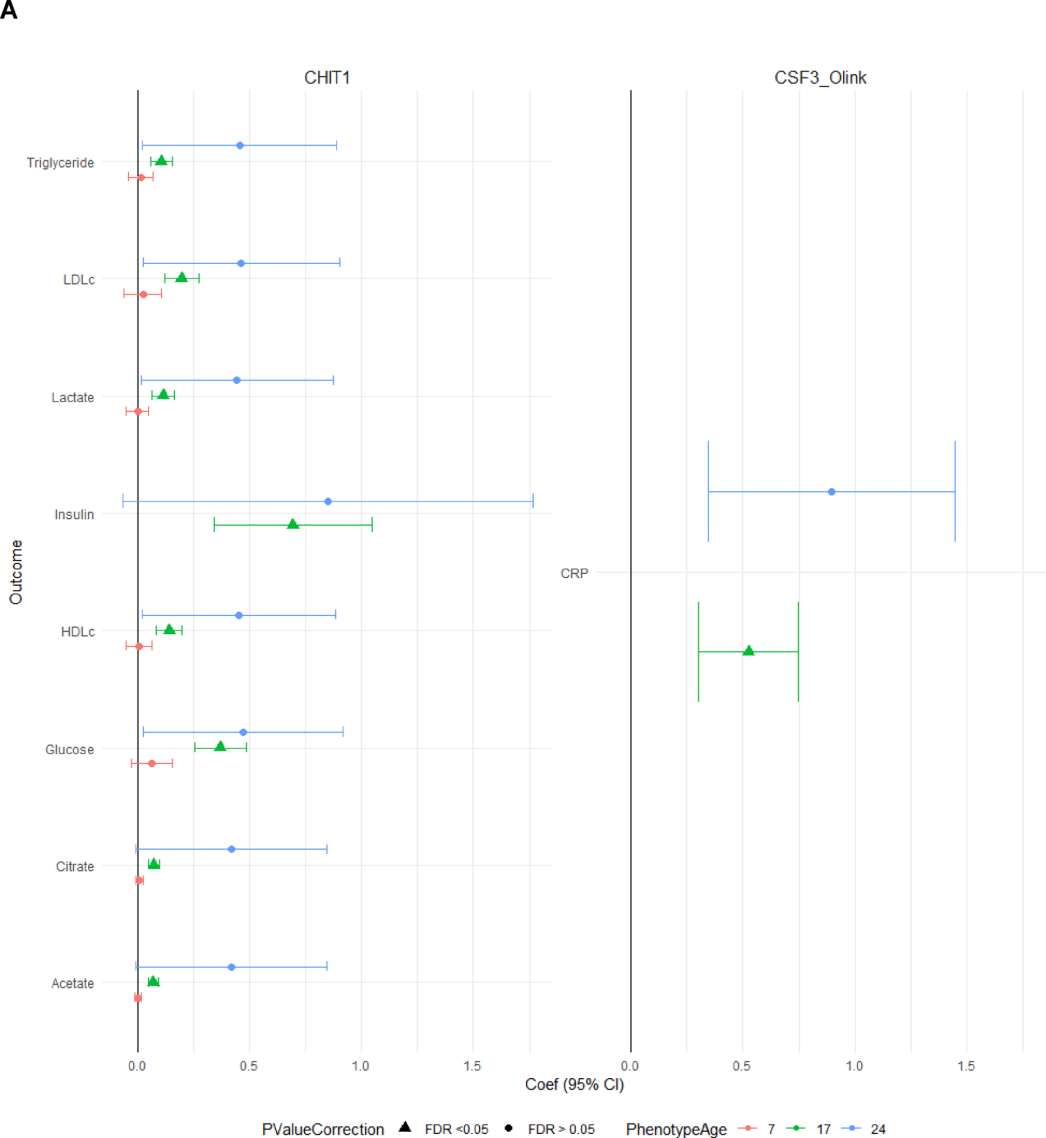

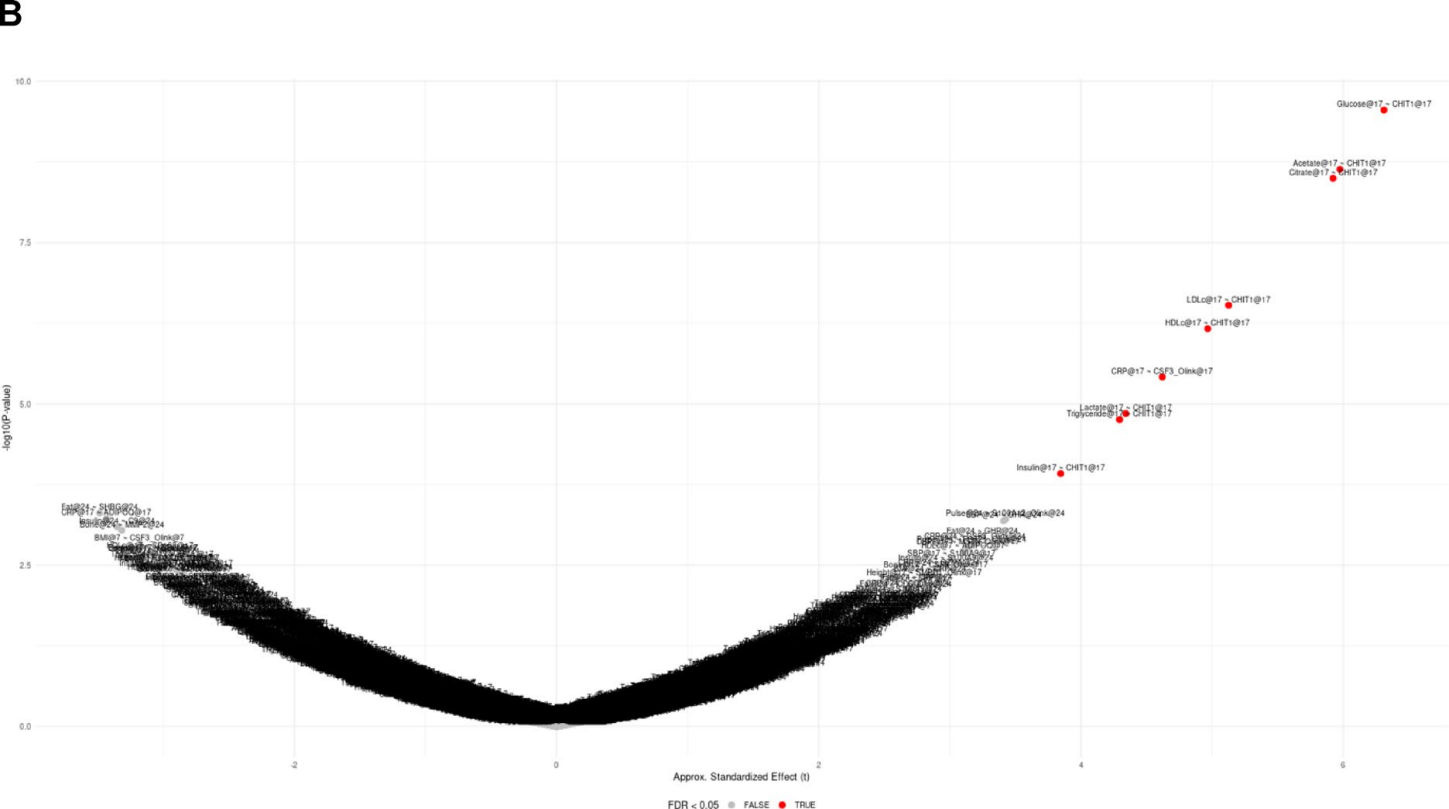
Episcores and outcomes with cross-sectional associations at ages seven, 17, or 24 (FDR < 0.05). A: Forest plot. Values are reported as the effect size in outcome per a + 1SD increase in the episcore. B: Volcano plot with approximated standardized effect sizes (t value).

In models examining cross-sectional associations between 108 episcores measured and 17 physical health phenotypes measured at 17 years of age, we find that increased *CHIT1* is associated with elevated levels of eight of the phenotypes (Acetate, Citrate, Glucose, Lactate, HDLc, LDLc, Triglyceride), and *CSF1* is associated with the inflammatory biomarker *CRP (*coefficient = 0.53 mg/l, *P* = 3.8e-06, FDR < 0.05) (Fig. 1). No phenotype-episcore associations were observed in cross-sectional analyses at ages seven and 24 (FDR < 0.05). A few of the associations observed at age 17 were also observed at age 24 but did not survive p-value adjustment for all tests performed at age 24. These included five associations with the CHIT1 episcore with Triglyceride, LDLc, HDLc, Lactate, Glucose levels (p-value range:0.038–0.043) and the single CSF3 episcore association with CRP levels (p-value = 0.001).

### Episcores predict numerous adulthood phenotypes

**Fig. 2:**
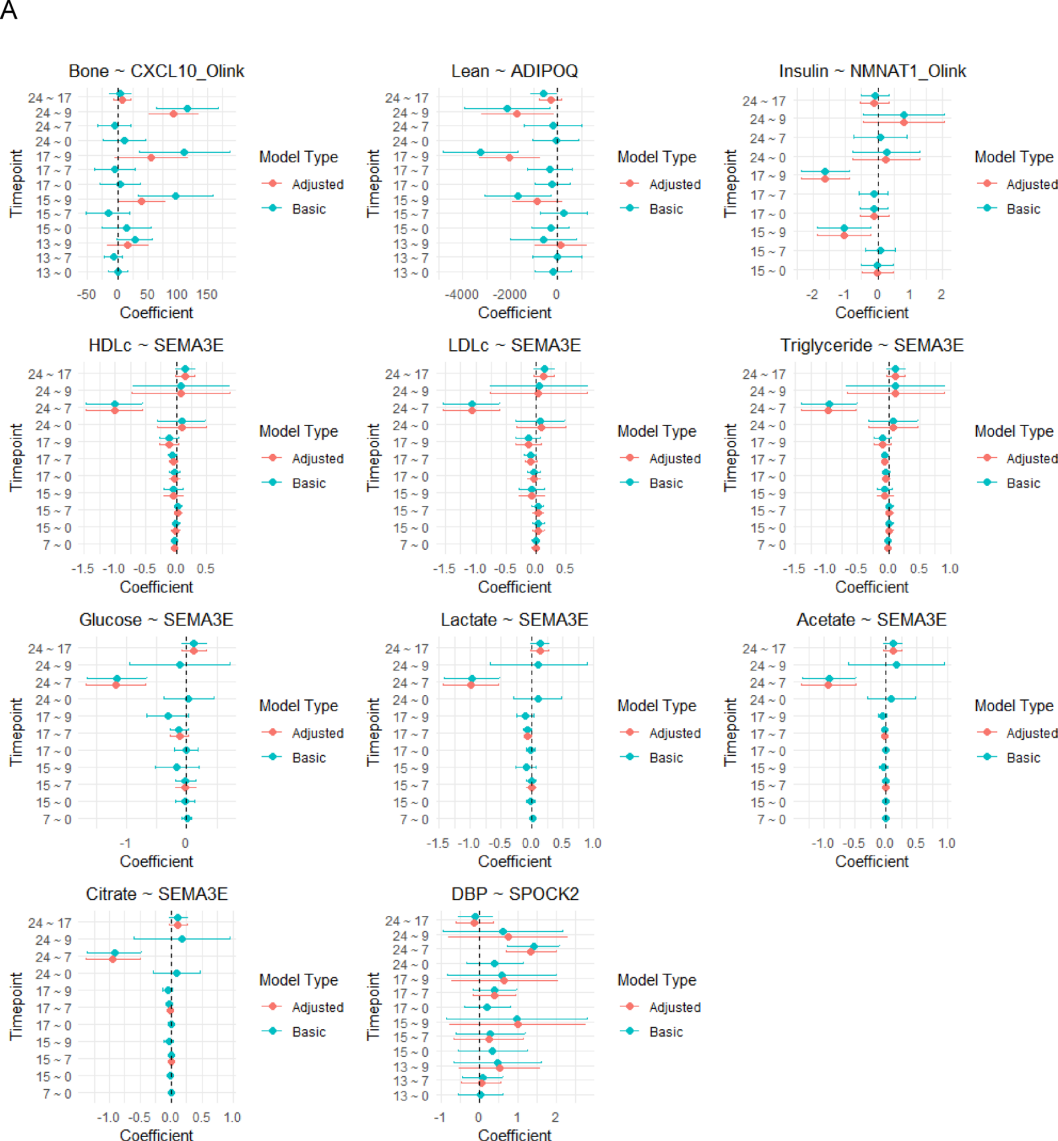

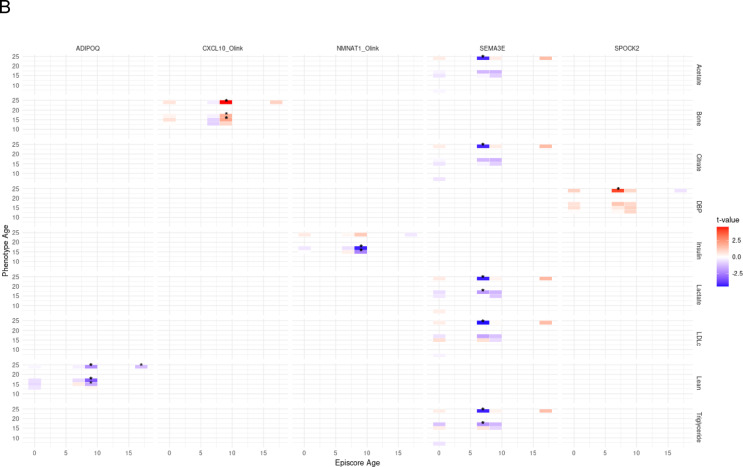
Episcores that predict future physical health phenotypes (FDR < 0.05 in at least one prospective model and *P* < 0.05 in the phenotype-adjusted prospective model). A: Forest plot. Values are reported as the effect size in outcome per a + 1SD increase in the episcore. B: Heatmap using approximated standardised effect sizes (t values).

In prospective models in which phenotypes are measured at least two years after episcores, we observe 11 associations (FDR < 0.05), all with nominal *p* < 0.05 in the phenotype-adjusted prospective model (Fig. 2, Supplemental Table 3). *SEMA3E* measured at age seven is associated with seven phenotypes measured at age 24 (Fig. 2). *SPOCK2* at age seven is associated with DBP at age 24 (phenotype-adjusted model coefficient = 1.34 mm/Hg, *P* = 6.9e-05). *NMNAT1* at age 9 is associated with reduced insulin at age 17 (phenotype-adjusted model coefficient = −1.63 mu/L, *P* = 1.9e-05), and shows directional consistency at age 15 (phenotype-adjusted model coefficient = −1.04mu/L, *P* = 0.014). *CXCL10* at age nine is associated with bone mass at age 24 (phenotype-adjusted model coefficient = 92.9 g, *P* = 5.7 e-06), and directionally consistent at ages 15 (phenotype-adjusted model coefficient = 39.9 g, *P* = 0.04). *ADIPOQ* at age 9 is associated with reduced lean mass at age 17 (phenotype-adjusted model coefficient = −2,057 g, *P* = 0.001) and is directionally consistent at age 24 (phenotype-adjusted model coefficient = −1,715 g, *P* = 0.03) (Supplementary Table 3).

### Causal effects of proteins on physical health risk factors

Having identified evidence that some episcores are associated with concurrent and future disease risk phenotypes, we examined whether the identified proteins may have causal effects on these phenotypes using two-sample Mendelian randomization (2SMR). Of the 20 episcore-phenotype pairs with cross-sectional or prospective associations, 16 had suitable genetic instruments (median number of instruments per protein: 3, Range: 2–4). However, none of these analyses indicated sufficient evidence of a causal effect of the target protein on the related physical health risk factor phenotype (2SMR *p* > 0.05). Results are summarised in Supplementary Table 4.

## Discussion

Here, we examined the capacity of DNAm protein abundance proxies (episcores) to explain variance in physical health phenotypes cross-sectionally and prospectively. We found 9 cross-sectional associations, and 11 prospective associations. Of these, most (8/9) cross-sectional associations were related to the protein *CHIT1*, and most of the prospective associations were related to the protein *SEMA3E* (8/11). A small number of these associations demonstrated directional consistency at multiple timepoints. There was no evidence from two-sample Mendelian randomization (2SMR) to suggest that any of the underlying proteins were causally related to the associated phenotype.

In the cross-sectional analyses, we find 8 associations between *CHIT1* and physical health risk factors, and in the predictive analysis we find seven associations between *SEMA3E* and physical health risk factors. These associations are consistent with previous findings that both CHIT1 and SEMA3E are associated with atherosclerosis and obesity^22^^–25^, which have been linked to the episcore-associated phenotypes (notably cholesterols and glucose)^26–28^. We also note that these *SEMA3E* associations are only found at one time point, and as such may not be a particularly robust biomarker for these physical health risk phenotypes.

We observed fewer associations with other episcores. However, these associations also appear to be consistent with the existing literature. We observed an association between *CXCL10* and future bone mass. Indeed, *CXCL10* has previously been associated with peak bone mass^29^, osteoclast differentiation^30^, and osteoclast formation^31^. Both *CCL22* and *NMNAT1* were associated with insulin levels. There is a body of literature linking *CCL22* to diabetes^32,33^ and *NMNAT1* is an adipocyte involved in NAD + biosynthesis^34^ which has been implicated in insulin resistance^35^. Finally, *CSF-3* was associated with the inflammatory biomarker *CRP*. We note *CSF-3* is one of six *IL6* protein domain homologues present in humans (identified via STRING^36^ and SMART^37^, and as such may be picking up on a relationship between *IL6* and *CRP* as inflammatory biomarkers^38^. In summary, there is a strong body of previous evidence suggesting that our phenotype-episcore associations are biologically feasible (including some evidence that these associations may be causal). However, we also note that many of the proteins which are evaluated here likely have strong associations in the literature with our phenotypes of interest yet fail to replicate in our analysis. As such, we do not further consider the biological role of the associations we find here.

We observed a surprising lack of consistency in direction of association effects across age spans (Fig. 2). These could indicate real biological differences, e.g. developmental differences across distinct age spans such as sexual maturation across puberty or physical maturation between adolescence and adulthood. However, some inconsistencies could be spuriously due to technical noise or unobserved confounding. Such effects would not need to be strong given that most episcores explain only a small portion of variance in the protein of interest. This is consistent with the fact that some of our strongest and most consistently observed episcore associations (CRP with CSF3 and Bone Mass with CXCL11) involve episcores with strong associations to their underlying target proteins (CSF3 R: 0.34 and CXCL10_Olink R: 0.23).

We calculated the intraclass correlation for each phenotype and found large differences in between phenotypes (physical measures: Pulse, DBP, and SBP all had ICC < 0.10), whilst blood-based measures such as HDLc, LDLc, and Triglycerides demonstrated much larger ICC values (range: 0.31–0.58). This suggests that the physical measures showed little stability across individuals over time, with most of the variance attributable to within-person fluctuations or measurement noise, limiting their ability to capture consistent between-person differences. In contrast, the higher ICCs observed for the blood-based measures indicate greater between-person stability, meaning that individuals tended to maintain their relative ranking across time. As a result, associations with early-life exposures were more detectable for the blood-based traits, whereas the low reliability of the physical measures likely attenuated any true associations and reduced statistical power.

Using Mendelian randomization, we did not observe any evidence of causal effects of proteins on physical health risk factors suggested by the episcore associations we observed. However, this is perhaps unsurprising given that the episcores were generated using protein abundance data with genetic variation regressed out. As such, the episcore associations we observed are not reflective of genetically driven changes in protein variation, which is precisely what Mendelian randomization uses to evaluate causality.

In comparison to the study by Gadd et al. which reported 130 associations between 109 episcores and 12 binary (present or not) morbidities (1,308 tests), we observed only 20 associations even though performed an even larger number of tests (1,836 tests between 108 episcores and 17 continuous physical health risk factor outcomes). There are a few plausible reasons for this difference. One is that Gadd et al. investigated well-specified disease as outcomes, whilst we investigated risk factors for disease as outcomes. Another plausible reason is that the Gadd et al. population was much older (59–73 years of age) than our population (7–24 years of age), so outcomes were likely less pronounced in our population. Finally, we also note that the Gadd episcores explained larger percentages of proteomic variance in their older populations compared to our younger population, as we have previously shown^7^.

In summary, we set out to test if DNAm proxies of protein expression trained in older populations with a high burden of well-defined disease are able to capture milder physical health phenotypic variance observed throughout childhood and early adulthood. We report evidence consistent with the literature that these proxies can predict a number of phenotypic changes throughout the early life course both novel and previously discovered. We suspect that it would be insightful for future analyses to compare phenotype-episcore associations discovered in our analyses with associations with directly measured protein abundance. We hypothesise that direct measurements may have stronger associations with concurrently measured phenotypes, especially with highly dynamic metabolites. Meanwhile, it is possible that episcores, given the overall stability of the methylome, will capture longer-term protein abundance trends that are more indicative of future health phenotypes.

Future analyses may wish to incorporate more complex modelling strategies such as longitudinal growth curve modelling to better evaluate the relationship between episcores and phenotypes over time. Strengths of this study include that we provide one of the largest analyses of episcore associations with physical health risk factors throughout the early life course, both cross-sectionally and prospectively using data collected prospectively in a well-characterised longitudinal birth cohort. Limitations of our study include the near exclusively white European demographics of both the datasets in which the episcores were trained and evaluated. We also note that the individuals included in the ARIES subset are not representative of the larger ALSPAC cohort, with greater maternal education, and lower rates of maternal smoking for example (Table 1). Sample sizes at some age groups were relatively small and possibly underpowered for some episcores and outcomes. We recognise that the data analysed may present with missingness at random, and as such reduces statistical power and may limit generalisability. As such, our biomarker discovery findings should be interpreted cautiously and validated in external datasets.

## Supplementary Information

Below is the link to the electronic supplementary material.

Supplementary Material 1

Supplementary Material 2

Supplementary Material 3

Supplementary Material 4

## Data Availability

The study website contains details of all the data that is available through a fully searchable data dictionary https://www.bristol.ac.uk/alspac/researchers/our-data/.
