# Supplementary material for: Associations of DNA methylation estimators of protein abundance with concurrent and future physical health risk factors
Source: Sci Rep. 2025 Dec 10;16:2054. doi: 10.1038/s41598-025-31843-z (PMC12808646; doi:10.1038/s41598-025-31843-z)
Supplement: Supplementary file 2 — Supplementary Material 2 [file 41598_2025_31843_MOESM2_ESM.docx]

Note:

- ‘Age’ represents the Age at which the Phenotype was measured, and the age at which the DNAm was measured for episcore calculation.
  - E.g Var24_FifteenDNAm means variables (phenotypes) measures at age 24, and DNAm measured at age 15.
- ‘Var’ represents the phenotype of interest used in the model.
- Mean (and StdError) is the mean (and standard error) measurement of the phenotype of interest at this time point
- SampleN is the number of non-NA values reported at this time point for both episcore and phenotype.

| **Age** | **Var** | **Mean** | **StdError** | **SampleN** |
| --- | --- | --- | --- | --- |
| Var24_FifteenDNAm | Pulse | 67 | 0.248 | 1754 |
| Var24_NineDNAm | Pulse | 68.1 | 0.696 | 196 |
| Var24_SevenDNAm | Pulse | 66.5 | 0.406 | 613 |
| Var24_BirthDNAm | Pulse | 66.7 | 0.428 | 572 |
| Var24_FifteenDNAm | SBP | 116 | 0.267 | 1754 |
| Var24_NineDNAm | SBP | 118 | 0.841 | 196 |
| Var24_SevenDNAm | SBP | 117 | 0.449 | 613 |
| Var24_BirthDNAm | SBP | 117 | 0.466 | 572 |
| Var24_FifteenDNAm | DBP | 66.9 | 0.188 | 1754 |
| Var24_NineDNAm | DBP | 67.7 | 0.574 | 196 |
| Var24_SevenDNAm | DBP | 66.8 | 0.296 | 613 |
| Var24_BirthDNAm | DBP | 67 | 0.313 | 572 |
| Var24_FifteenDNAm | Fat | 22800 | 245 | 1691 |
| Var24_NineDNAm | Fat | 23700 | 832 | 192 |
| Var24_SevenDNAm | Fat | 22300 | 387 | 591 |
| Var24_BirthDNAm | Fat | 22300 | 409 | 548 |
| Var24_FifteenDNAm | Lean | 47900 | 238 | 1691 |
| Var24_NineDNAm | Lean | 47800 | 671 | 192 |
| Var24_SevenDNAm | Lean | 48000 | 400 | 591 |
| Var24_BirthDNAm | Lean | 48000 | 414 | 548 |
| Var24_FifteenDNAm | Bone | 2660 | 11.9 | 1691 |
| Var24_NineDNAm | Bone | 2640 | 33.7 | 192 |
| Var24_SevenDNAm | Bone | 2670 | 20.1 | 591 |
| Var24_BirthDNAm | Bone | 2670 | 20.8 | 548 |
| Var24_FifteenDNAm | BMI | 24.7 | 0.113 | 1737 |
| Var24_NineDNAm | BMI | 25.1 | 0.371 | 194 |
| Var24_SevenDNAm | BMI | 24.5 | 0.186 | 608 |
| Var24_BirthDNAm | BMI | 24.5 | 0.197 | 567 |
| Var24_FifteenDNAm | Height | 172 | 2.23 | 1739 |
| Var24_NineDNAm | Height | 172 | 6.59 | 194 |
| Var24_SevenDNAm | Height | 172 | 3.71 | 609 |
| Var24_BirthDNAm | Height | 173 | 3.82 | 568 |
| Var24_FifteenDNAm | Glucose | 3.92 | 0.00814 | 2855 |
| Var24_NineDNAm | Glucose | 3.96 | 0.0287 | 361 |
| Var24_SevenDNAm | Glucose | 3.95 | 0.017 | 969 |
| Var24_BirthDNAm | Glucose | 3.96 | 0.018 | 905 |
| Var24_FifteenDNAm | Lactate | 0.932 | 0.00832 | 2855 |
| Var24_NineDNAm | Lactate | 0.911 | 0.0189 | 361 |
| Var24_SevenDNAm | Lactate | 0.929 | 0.014 | 969 |
| Var24_BirthDNAm | Lactate | 0.935 | 0.0148 | 905 |
| Var24_FifteenDNAm | Citrate | 0.16 | 0.000464 | 2855 |
| Var24_NineDNAm | Citrate | 0.161 | 0.00148 | 361 |
| Var24_SevenDNAm | Citrate | 0.161 | 0.000834 | 969 |
| Var24_BirthDNAm | Citrate | 0.16 | 0.000862 | 905 |
| Var24_FifteenDNAm | Acetate | 0.0513 | 0.00064 | 2855 |
| Var24_NineDNAm | Acetate | 0.0514 | 0.00192 | 361 |
| Var24_SevenDNAm | Acetate | 0.051 | 0.00107 | 969 |
| Var24_BirthDNAm | Acetate | 0.0509 | 0.00114 | 905 |
| Var24_FifteenDNAm | HDL | 1.54 | 0.0078 | 2857 |
| Var24_NineDNAm | HDL | 1.53 | 0.0209 | 361 |
| Var24_SevenDNAm | HDL | 1.55 | 0.0135 | 969 |
| Var24_BirthDNAm | HDL | 1.56 | 0.0141 | 905 |
| Var24_FifteenDNAm | LDL | 2.42 | 0.0142 | 2857 |
| Var24_NineDNAm | LDL | 2.4 | 0.038 | 361 |
| Var24_SevenDNAm | LDL | 2.4 | 0.0243 | 969 |
| Var24_BirthDNAm | LDL | 2.38 | 0.0251 | 905 |
| Var24_FifteenDNAm | Triglycerides | 0.951 | 0.00881 | 2857 |
| Var24_NineDNAm | Triglycerides | 0.992 | 0.0302 | 361 |
| Var24_SevenDNAm | Triglycerides | 0.963 | 0.0178 | 969 |
| Var24_BirthDNAm | Triglycerides | 0.953 | 0.0183 | 905 |
| Var24_FifteenDNAm | Insulin | 9.46 | 0.17 | 2857 |
| Var24_NineDNAm | Insulin | 9.59 | 0.407 | 361 |
| Var24_SevenDNAm | Insulin | 9.25 | 0.311 | 969 |
| Var24_BirthDNAm | Insulin | 9.32 | 0.332 | 905 |
| Var24_FifteenDNAm | CRP | 2.05 | 0.0733 | 2857 |
| Var24_NineDNAm | CRP | 2.09 | 0.157 | 361 |
| Var24_SevenDNAm | CRP | 2.13 | 0.138 | 969 |
| Var24_BirthDNAm | CRP | 2.12 | 0.142 | 905 |
| Var17_FifteenDNAm | Height | 172 | 0.183 | 2658 |
| Var17_NineDNAm | Height | 172 | 0.583 | 248 |
| Var17_SevenDNAm | Height | 172 | 0.32 | 829 |
| Var17_BirthDNAm | Height | 173 | 0.327 | 779 |
| Var17_FifteenDNAm | BMI | 22.7 | 0.0759 | 2657 |
| Var17_NineDNAm | BMI | 22.9 | 0.273 | 248 |
| Var17_SevenDNAm | BMI | 22.6 | 0.133 | 829 |
| Var17_BirthDNAm | BMI | 22.6 | 0.138 | 779 |
| Var17_FifteenDNAm | Bone | 2770 | 10.4 | 2614 |
| Var17_NineDNAm | Bone | 2750 | 33.1 | 244 |
| Var17_SevenDNAm | Bone | 2790 | 18.9 | 820 |
| Var17_BirthDNAm | Bone | 2790 | 19.6 | 769 |
| Var17_FifteenDNAm | Lean | 46300 | 198 | 2614 |
| Var17_NineDNAm | Lean | 45700 | 634 | 244 |
| Var17_SevenDNAm | Lean | 46600 | 348 | 820 |
| Var17_BirthDNAm | Lean | 46500 | 360 | 769 |
| Var17_FifteenDNAm | Fat | 17700 | 196 | 2614 |
| Var17_NineDNAm | Fat | 18700 | 701 | 244 |
| Var17_SevenDNAm | Fat | 17700 | 350 | 820 |
| Var17_BirthDNAm | Fat | 17900 | 361 | 769 |
| Var17_FifteenDNAm | Pulse | 65 | 0.212 | 2545 |
| Var17_NineDNAm | Pulse | 65.3 | 0.74 | 232 |
| Var17_SevenDNAm | Pulse | 64 | 0.363 | 791 |
| Var17_BirthDNAm | Pulse | 64.1 | 0.373 | 743 |
| Var17_FifteenDNAm | DBP | 63.9 | 0.147 | 2545 |
| Var17_NineDNAm | DBP | 63.6 | 0.453 | 232 |
| Var17_SevenDNAm | DBP | 63.4 | 0.24 | 791 |
| Var17_BirthDNAm | DBP | 63.3 | 0.25 | 743 |
| Var17_FifteenDNAm | SBP | 120 | 0.222 | 2545 |
| Var17_NineDNAm | SBP | 119 | 0.703 | 232 |
| Var17_SevenDNAm | SBP | 119 | 0.396 | 791 |
| Var17_BirthDNAm | SBP | 119 | 0.411 | 743 |
| Var17_FifteenDNAm | Acetate | 0.0434 | 0.000437 | 2855 |
| Var17_NineDNAm | Acetate | 0.0418 | 0.000541 | 361 |
| Var17_SevenDNAm | Acetate | 0.0442 | 0.000842 | 969 |
| Var17_BirthDNAm | Acetate | 0.0454 | 0.00115 | 905 |
| Var17_FifteenDNAm | Citrate | 0.0939 | 0.000417 | 2855 |
| Var17_NineDNAm | Citrate | 0.0931 | 0.00119 | 361 |
| Var17_SevenDNAm | Citrate | 0.0945 | 0.000702 | 969 |
| Var17_BirthDNAm | Citrate | 0.0945 | 0.000722 | 905 |
| Var17_FifteenDNAm | Lactate | 0.99 | 0.00855 | 2855 |
| Var17_NineDNAm | Lactate | 1.03 | 0.0259 | 361 |
| Var17_SevenDNAm | Lactate | 0.992 | 0.0162 | 969 |
| Var17_BirthDNAm | Lactate | 0.993 | 0.0164 | 905 |
| Var17_FifteenDNAm | Glucose | 4.15 | 0.00997 | 2855 |
| Var17_NineDNAm | Glucose | 4.16 | 0.0312 | 361 |
| Var17_SevenDNAm | Glucose | 4.15 | 0.0136 | 969 |
| Var17_BirthDNAm | Glucose | 4.14 | 0.014 | 905 |
| Var17_FifteenDNAm | CRP | 1.57 | 0.0774 | 2857 |
| Var17_NineDNAm | CRP | 1.44 | 0.125 | 361 |
| Var17_SevenDNAm | CRP | 1.44 | 0.107 | 969 |
| Var17_BirthDNAm | CRP | 1.39 | 0.104 | 905 |
| Var17_FifteenDNAm | Insulin | 8.19 | 0.114 | 2857 |
| Var17_NineDNAm | Insulin | 8.65 | 0.316 | 361 |
| Var17_SevenDNAm | Insulin | 7.89 | 0.174 | 969 |
| Var17_BirthDNAm | Insulin | 7.81 | 0.168 | 905 |
| Var17_FifteenDNAm | Triglycerides | 0.839 | 0.00673 | 2857 |
| Var17_NineDNAm | Triglycerides | 0.877 | 0.024 | 361 |
| Var17_SevenDNAm | Triglycerides | 0.847 | 0.0128 | 969 |
| Var17_BirthDNAm | Triglycerides | 0.85 | 0.0134 | 905 |
| Var17_FifteenDNAm | LDL | 2.1 | 0.0113 | 2857 |
| Var17_NineDNAm | LDL | 2.08 | 0.0315 | 361 |
| Var17_SevenDNAm | LDL | 2.09 | 0.0202 | 969 |
| Var17_BirthDNAm | LDL | 2.08 | 0.0209 | 905 |
| Var17_FifteenDNAm | HDL | 1.27 | 0.00551 | 2857 |
| Var17_NineDNAm | HDL | 1.26 | 0.0164 | 361 |
| Var17_SevenDNAm | HDL | 1.26 | 0.00963 | 969 |
| Var17_BirthDNAm | HDL | 1.26 | 0.01 | 905 |
| Var15_NineDNAm | Bone | 2510 | 27.4 | 260 |
| Var15_SevenDNAm | Bone | 2540 | 15.9 | 870 |
| Var15_BirthDNAm | Bone | 2540 | 16.5 | 812 |
| Var15_NineDNAm | Lean | 43100 | 503 | 260 |
| Var15_SevenDNAm | Lean | 43800 | 285 | 870 |
| Var15_BirthDNAm | Lean | 43700 | 296 | 812 |
| Var15_NineDNAm | Fat | 15700 | 569 | 260 |
| Var15_SevenDNAm | Fat | 15200 | 302 | 870 |
| Var15_BirthDNAm | Fat | 15400 | 312 | 812 |
| Var15_NineDNAm | Pulse | 74.3 | 0.936 | 260 |
| Var15_SevenDNAm | Pulse | 73.7 | 0.475 | 869 |
| Var15_BirthDNAm | Pulse | 74 | 0.498 | 805 |
| Var15_NineDNAm | DBP | 67.5 | 0.595 | 260 |
| Var15_SevenDNAm | DBP | 67 | 0.337 | 869 |
| Var15_BirthDNAm | DBP | 67.1 | 0.348 | 805 |
| Var15_NineDNAm | SBP | 125 | 0.818 | 260 |
| Var15_SevenDNAm | SBP | 123 | 0.435 | 869 |
| Var15_BirthDNAm | SBP | 123 | 0.447 | 805 |
| Var15_NineDNAm | Acetate | 0.0421 | 0.000485 | 361 |
| Var15_SevenDNAm | Acetate | 0.0431 | 0.000311 | 969 |
| Var15_BirthDNAm | Acetate | 0.0432 | 0.000329 | 905 |
| Var15_NineDNAm | Citrate | 0.112 | 0.00116 | 361 |
| Var15_SevenDNAm | Citrate | 0.112 | 0.000748 | 969 |
| Var15_BirthDNAm | Citrate | 0.111 | 0.000774 | 905 |
| Var15_NineDNAm | Lactate | 1.28 | 0.0331 | 361 |
| Var15_SevenDNAm | Lactate | 1.27 | 0.0202 | 969 |
| Var15_BirthDNAm | Lactate | 1.27 | 0.0208 | 905 |
| Var15_NineDNAm | Glucose | 4.29 | 0.0178 | 361 |
| Var15_SevenDNAm | Glucose | 4.31 | 0.0104 | 969 |
| Var15_BirthDNAm | Glucose | 4.31 | 0.0105 | 905 |
| Var15_NineDNAm | CRP | 1.18 | 0.227 | 361 |
| Var15_SevenDNAm | CRP | 1.14 | 0.127 | 969 |
| Var15_BirthDNAm | CRP | 1.21 | 0.14 | 905 |
| Var15_NineDNAm | Insulin | 10.4 | 0.258 | 361 |
| Var15_SevenDNAm | Insulin | 9.86 | 0.165 | 969 |
| Var15_BirthDNAm | Insulin | 9.89 | 0.166 | 905 |
| Var15_NineDNAm | Triglycerides | 0.832 | 0.0188 | 361 |
| Var15_SevenDNAm | Triglycerides | 0.831 | 0.0119 | 969 |
| Var15_BirthDNAm | Triglycerides | 0.825 | 0.0117 | 905 |
| Var15_NineDNAm | LDL | 2.12 | 0.031 | 361 |
| Var15_SevenDNAm | LDL | 2.06 | 0.0177 | 969 |
| Var15_BirthDNAm | LDL | 2.06 | 0.0185 | 905 |
| Var15_NineDNAm | HDL | 1.29 | 0.0159 | 361 |
| Var15_SevenDNAm | HDL | 1.27 | 0.00945 | 969 |
| Var15_BirthDNAm | HDL | 1.28 | 0.00989 | 905 |
| Var13_NineDNAm | Height | 164 | 0.439 | 296 |
| Var13_SevenDNAm | Height | 164 | 0.259 | 885 |
| Var13_BirthDNAm | Height | 164 | 0.268 | 825 |
| Var13_NineDNAm | BMI | 20.3 | 0.196 | 296 |
| Var13_SevenDNAm | BMI | 20.2 | 0.111 | 882 |
| Var13_BirthDNAm | BMI | 20.3 | 0.115 | 824 |
| Var13_NineDNAm | Bone | 2110 | 22.8 | 293 |
| Var13_SevenDNAm | Bone | 2130 | 13.8 | 875 |
| Var13_BirthDNAm | Bone | 2140 | 14.3 | 817 |
| Var13_NineDNAm | Lean | 38000 | 364 | 293 |
| Var13_SevenDNAm | Lean | 38600 | 219 | 875 |
| Var13_BirthDNAm | Lean | 38600 | 228 | 817 |
| Var13_NineDNAm | Fat | 13900 | 474 | 293 |
| Var13_SevenDNAm | Fat | 13300 | 260 | 875 |
| Var13_BirthDNAm | Fat | 13500 | 269 | 817 |
| Var13_NineDNAm | Pulse | 70.5 | 0.757 | 258 |
| Var13_SevenDNAm | Pulse | 70.7 | 0.405 | 767 |
| Var13_BirthDNAm | Pulse | 70.7 | 0.422 | 719 |
| Var13_NineDNAm | DBP | 57.8 | 0.383 | 258 |
| Var13_SevenDNAm | DBP | 58.1 | 0.232 | 767 |
| Var13_BirthDNAm | DBP | 58.2 | 0.242 | 719 |
| Var13_NineDNAm | SBP | 107 | 0.6 | 258 |
| Var13_SevenDNAm | SBP | 108 | 0.375 | 767 |
| Var13_BirthDNAm | SBP | 108 | 0.392 | 719 |
| Var10_SevenDNAm | Height | 144 | 0.214 | 914 |
| Var10_BirthDNAm | Height | 145 | 0.223 | 853 |
| Var10_SevenDNAm | BMI | 18.2 | 0.103 | 914 |
| Var10_BirthDNAm | BMI | 18.2 | 0.107 | 853 |
| Var7_BirthDNAm | Height | 126 | 0.173 | 902 |
| Var7_BirthDNAm | BMI | 16.2 | 0.07 | 902 |
| Var7_BirthDNAm | Pulse | 82.9 | 0.363 | 899 |
| Var7_BirthDNAm | Acetate | 0.0592 | 0.000819 | 905 |
| Var7_BirthDNAm | Citrate | 0.127 | 0.000847 | 905 |
| Var7_BirthDNAm | Lactate | 1.36 | 0.016 | 905 |
| Var7_BirthDNAm | Glucose | 4.19 | 0.0164 | 905 |
| Var7_BirthDNAm | Triglycerides | 1.06 | 0.0184 | 905 |
| Var7_BirthDNAm | LDL | 2.23 | 0.0185 | 905 |
| Var7_BirthDNAm | HDL | 1.52 | 0.0105 | 905 |
