# Supplementary material for: Associations of DNA methylation estimators of protein abundance with concurrent and future physical health risk factors
Source: Sci Rep. 2025 Dec 10;16:2054. doi: 10.1038/s41598-025-31843-z (PMC12808646; doi:10.1038/s41598-025-31843-z)
Supplement: Supplementary file 3 — Supplementary Material 3 [file 41598_2025_31843_MOESM3_ESM.docx]

Supplementary Table 4 – two sample mendelian randomisation results for phenotype-Episcore associations discovered in ALSPAC.

Note – NA values are present in Q values in cases where there are not enough instruments to calculate this statistic

| **outcome** | **exposure** | **nsnp** | **SNP** | **b** | **pval** | **Q** | **intercept** | **intercept_pval** | **Method** | **Cis** |
| --- | --- | --- | --- | --- | --- | --- | --- | --- | --- | --- |
| Acetate | Chitotriosidase-1 \|\| id:prot-a-543 | 3 | rs12411260; rs7072852; rs872583 | -0.00149374543728934 | 0.834844844985579 | 4.8918824092069 | NA | NA | Inverse variance weighted | FALSE |
| Acetate | Chitotriosidase-1 \|\| id:prot-a-543 | 3 | rs12411260; rs7072852; rs872583 | 0.00898904861450295 | 0.40360339087775 | 0.0494108134761568 | -0.00879709665567606 | 0.271538284411191 | MR Egger | FALSE |
| Acetate | Chitotriosidase-1 \|\| id:prot-a-543 | 3 | rs12411260; rs7072852; rs872583 | -0.0412920295969483 | 0.261376554318005 | NA | NA | NA | Simple mode | FALSE |
| Acetate | Chitotriosidase-1 \|\| id:prot-a-543 | 1 | rs12411260 | -0.0348918787222523 | 0.150957338951955 | NA | NA | NA | Wald ratio | FALSE |
| Acetate | Chitotriosidase-1 \|\| id:prot-a-543 | 1 | rs7072852 | -0.0528030361757106 | 0.0888575388871701 | NA | NA | NA | Wald ratio | FALSE |
| Acetate | Chitotriosidase-1 \|\| id:prot-a-543 | 1 | rs872583 | 0.000951939537424877 | 0.840109139938182 | NA | NA | NA | Wald ratio | FALSE |
| Acetate | Chitotriosidase-1 \|\| id:prot-a-543 | 3 | rs12411260; rs7072852; rs872583 | -0.00102606871516887 | 0.827758345412374 | NA | NA | NA | Weighted median | FALSE |
| Acetate | Chitotriosidase-1 \|\| id:prot-a-543 | 3 | rs12411260; rs7072852; rs872583 | 0.000676639699423623 | 0.906594664914028 | NA | NA | NA | Weighted mode | FALSE |
| Lactate | Chitotriosidase-1 \|\| id:prot-a-543 | 3 | rs12411260; rs7072852; rs872583 | -0.00769517426958702 | 0.0911209933940964 | 0.89257499702451 | NA | NA | Inverse variance weighted | FALSE |
| Lactate | Chitotriosidase-1 \|\| id:prot-a-543 | 3 | rs12411260; rs7072852; rs872583 | -0.0076308956935326 | 0.452580689482288 | 0.892390825430686 | -5.39439349449383E-05 | 0.991360973886158 | MR Egger | FALSE |
| Lactate | Chitotriosidase-1 \|\| id:prot-a-543 | 3 | rs12411260; rs7072852; rs872583 | -0.00399294195791668 | 0.711261230285658 | NA | NA | NA | Simple mode | FALSE |
| Lactate | Chitotriosidase-1 \|\| id:prot-a-543 | 1 | rs12411260 | 0.0079122360584732 | 0.743242583455185 | NA | NA | NA | Wald ratio | FALSE |
| Lactate | Chitotriosidase-1 \|\| id:prot-a-543 | 1 | rs7072852 | -0.0289569121447028 | 0.348092407702525 | NA | NA | NA | Wald ratio | FALSE |
| Lactate | Chitotriosidase-1 \|\| id:prot-a-543 | 1 | rs872583 | -0.00779256965944272 | 0.0966883595008454 | NA | NA | NA | Wald ratio | FALSE |
| Lactate | Chitotriosidase-1 \|\| id:prot-a-543 | 3 | rs12411260; rs7072852; rs872583 | -0.00756236350338199 | 0.100208937807789 | NA | NA | NA | Weighted median | FALSE |
| Lactate | Chitotriosidase-1 \|\| id:prot-a-543 | 3 | rs12411260; rs7072852; rs872583 | -0.00765856783030862 | 0.245852436595836 | NA | NA | NA | Weighted mode | FALSE |
| Fasting glucose | Chitotriosidase-1 \|\| id:prot-a-543 | 3 | rs12411260; rs7072852; rs872583 | 0.000923588436314792 | 0.667256331118943 | 2.23731368554771 | NA | NA | Inverse variance weighted | FALSE |
| Fasting glucose | Chitotriosidase-1 \|\| id:prot-a-543 | 3 | rs12411260; rs7072852; rs872583 | 0.00157251698328161 | 0.776061412659282 | 2.14251654711415 | -0.00054099242829739 | 0.868013351154848 | MR Egger | FALSE |
| Fasting glucose | Chitotriosidase-1 \|\| id:prot-a-543 | 3 | rs12411260; rs7072852; rs872583 | 0.00438294118609828 | 0.430425897913662 | NA | NA | NA | Simple mode | FALSE |
| Fasting glucose | Chitotriosidase-1 \|\| id:prot-a-543 | 1 | rs12411260 | 0.00866269626421224 | 0.399729035501385 | NA | NA | NA | Wald ratio | FALSE |
| Fasting glucose | Chitotriosidase-1 \|\| id:prot-a-543 | 1 | rs7072852 | -0.0174418604651163 | 0.219720102570242 | NA | NA | NA | Wald ratio | FALSE |
| Fasting glucose | Chitotriosidase-1 \|\| id:prot-a-543 | 1 | rs872583 | 0.00100163904571116 | 0.632464543594736 | NA | NA | NA | Wald ratio | FALSE |
| Fasting glucose | Chitotriosidase-1 \|\| id:prot-a-543 | 3 | rs12411260; rs7072852; rs872583 | 0.00115002654013528 | 0.585528379246566 | NA | NA | NA | Weighted median | FALSE |
| Fasting glucose | Chitotriosidase-1 \|\| id:prot-a-543 | 3 | rs12411260; rs7072852; rs872583 | 0.00128675649672758 | 0.603355425427149 | NA | NA | NA | Weighted mode | FALSE |
| Fasting insulin | Chitotriosidase-1 \|\| id:prot-a-543 | 3 | rs12411260; rs7072852; rs872583 | -0.00315336923811048 | 0.185825412207091 | 0.673560253537187 | NA | NA | Inverse variance weighted | FALSE |
| Fasting insulin | Chitotriosidase-1 \|\| id:prot-a-543 | 3 | rs12411260; rs7072852; rs872583 | -0.00422247732994059 | 0.434519683178402 | 0.486189648798559 | 0.000888954695176655 | 0.739933180215503 | MR Egger | FALSE |
| Fasting insulin | Chitotriosidase-1 \|\| id:prot-a-543 | 3 | rs12411260; rs7072852; rs872583 | -0.00410703589737324 | 0.481897531360057 | NA | NA | NA | Simple mode | FALSE |
| Fasting insulin | Chitotriosidase-1 \|\| id:prot-a-543 | 1 | rs12411260 | -0.00487276664861938 | 0.682472947499482 | NA | NA | NA | Wald ratio | FALSE |
| Fasting insulin | Chitotriosidase-1 \|\| id:prot-a-543 | 1 | rs7072852 | 0.0103359173126615 | 0.538300749018723 | NA | NA | NA | Wald ratio | FALSE |
| Fasting insulin | Chitotriosidase-1 \|\| id:prot-a-543 | 1 | rs872583 | -0.0033691495173921 | 0.170571316384161 | NA | NA | NA | Wald ratio | FALSE |
| Fasting insulin | Chitotriosidase-1 \|\| id:prot-a-543 | 3 | rs12411260; rs7072852; rs872583 | -0.00339989177747012 | 0.165079820913331 | NA | NA | NA | Weighted median | FALSE |
| Fasting insulin | Chitotriosidase-1 \|\| id:prot-a-543 | 3 | rs12411260; rs7072852; rs872583 | -0.00342560844391046 | 0.282907605106366 | NA | NA | NA | Weighted mode | FALSE |
| HDL cholesterol | Chitotriosidase-1 \|\| id:prot-a-543 | 3 | rs12411260; rs7072852; rs872583 | -0.00383839754669391 | 0.541115417376793 | 17.9826166251667 | NA | NA | Inverse variance weighted | FALSE |
| HDL cholesterol | Chitotriosidase-1 \|\| id:prot-a-543 | 3 | rs12411260; rs7072852; rs872583 | 0.00538309605251313 | 0.325750127427633 | 0.0846860512350203 | -0.00774215289477963 | 0.147767775546372 | MR Egger | FALSE |
| HDL cholesterol | Chitotriosidase-1 \|\| id:prot-a-543 | 3 | rs12411260; rs7072852; rs872583 | -0.0402324985519965 | 0.104227912432121 | NA | NA | NA | Simple mode | FALSE |
| HDL cholesterol | Chitotriosidase-1 \|\| id:prot-a-543 | 1 | rs12411260 | -0.0387697888467786 | 0.000475869927333336 | NA | NA | NA | Wald ratio | FALSE |
| HDL cholesterol | Chitotriosidase-1 \|\| id:prot-a-543 | 1 | rs7072852 | -0.0416415374677003 | 0.00346062704690918 | NA | NA | NA | Wald ratio | FALSE |
| HDL cholesterol | Chitotriosidase-1 \|\| id:prot-a-543 | 1 | rs872583 | -0.00165083773447459 | 0.444133255198335 | NA | NA | NA | Wald ratio | FALSE |
| HDL cholesterol | Chitotriosidase-1 \|\| id:prot-a-543 | 3 | rs12411260; rs7072852; rs872583 | -0.00337214226490341 | 0.104539483509818 | NA | NA | NA | Weighted median | FALSE |
| HDL cholesterol | Chitotriosidase-1 \|\| id:prot-a-543 | 3 | rs12411260; rs7072852; rs872583 | -0.00157297177410565 | 0.554899502530847 | NA | NA | NA | Weighted mode | FALSE |
| LDL cholesterol | Chitotriosidase-1 \|\| id:prot-a-543 | 3 | rs12411260; rs7072852; rs872583 | 0.000554662371944321 | 0.850346626450124 | 3.2956695416149 | NA | NA | Inverse variance weighted | FALSE |
| LDL cholesterol | Chitotriosidase-1 \|\| id:prot-a-543 | 3 | rs12411260; rs7072852; rs872583 | -0.00201531729013031 | 0.748229230006671 | 2.1330003019225 | 0.0021579232490384 | 0.595128476563044 | MR Egger | FALSE |
| LDL cholesterol | Chitotriosidase-1 \|\| id:prot-a-543 | 3 | rs12411260; rs7072852; rs872583 | -0.00131892634292423 | 0.831704000103182 | NA | NA | NA | Simple mode | FALSE |
| LDL cholesterol | Chitotriosidase-1 \|\| id:prot-a-543 | 1 | rs12411260 | -0.00259656199242014 | 0.830535037396056 | NA | NA | NA | Wald ratio | FALSE |
| LDL cholesterol | Chitotriosidase-1 \|\| id:prot-a-543 | 1 | rs7072852 | 0.0283366279069767 | 0.0689315323031655 | NA | NA | NA | Wald ratio | FALSE |
| LDL cholesterol | Chitotriosidase-1 \|\| id:prot-a-543 | 1 | rs872583 | 3.69928064104899E-05 | 0.987486292499672 | NA | NA | NA | Wald ratio | FALSE |
| LDL cholesterol | Chitotriosidase-1 \|\| id:prot-a-543 | 3 | rs12411260; rs7072852; rs872583 | -6.48202888821683E-06 | 0.997840667615842 | NA | NA | NA | Weighted median | FALSE |
| LDL cholesterol | Chitotriosidase-1 \|\| id:prot-a-543 | 3 | rs12411260; rs7072852; rs872583 | -7.73538448967277E-06 | 0.99779996902635 | NA | NA | NA | Weighted mode | FALSE |
| triglycerides | Chitotriosidase-1 \|\| id:prot-a-543 | 3 | rs12411260; rs7072852; rs872583 | 0.00404852082096224 | 0.597397529048614 | 24.6322321276269 | NA | NA | Inverse variance weighted | FALSE |
| triglycerides | Chitotriosidase-1 \|\| id:prot-a-543 | 3 | rs12411260; rs7072852; rs872583 | -0.00723132340268176 | 0.261699278743054 | 0.000381509067894167 | 0.00946964517480943 | 0.126577161980191 | MR Egger | FALSE |
| triglycerides | Chitotriosidase-1 \|\| id:prot-a-543 | 3 | rs12411260; rs7072852; rs872583 | 0.0490358205100619 | 0.0853521294027635 | NA | NA | NA | Simple mode | FALSE |
| triglycerides | Chitotriosidase-1 \|\| id:prot-a-543 | 1 | rs12411260 | 0.0438825663237683 | 0.000149381769574497 | NA | NA | NA | Wald ratio | FALSE |
| triglycerides | Chitotriosidase-1 \|\| id:prot-a-543 | 1 | rs7072852 | 0.0541511627906977 | 0.000264776786700033 | NA | NA | NA | Wald ratio | FALSE |
| triglycerides | Chitotriosidase-1 \|\| id:prot-a-543 | 1 | rs872583 | 0.0013926698233473 | 0.535873815486718 | NA | NA | NA | Wald ratio | FALSE |
| triglycerides | Chitotriosidase-1 \|\| id:prot-a-543 | 3 | rs12411260; rs7072852; rs872583 | 0.00323978179511836 | 0.152807397397421 | NA | NA | NA | Weighted median | FALSE |
| triglycerides | Chitotriosidase-1 \|\| id:prot-a-543 | 3 | rs12411260; rs7072852; rs872583 | 0.0013953793994271 | 0.61233869472585 | NA | NA | NA | Weighted mode | FALSE |
| Acetate | Chitotriosidase-1 \|\| id:prot-a-543 | 2 | rs12411260; rs872583 | -0.00035082626777748 | 0.958290665053122 | 2.09751787385932 | NA | NA | Inverse variance weighted | TRUE |
| Acetate | Chitotriosidase-1 \|\| id:prot-a-543 | 1 | rs12411260 | -0.0348918787222523 | 0.150957338951955 | NA | NA | NA | Wald ratio | TRUE |
| Acetate | Chitotriosidase-1 \|\| id:prot-a-543 | 1 | rs872583 | 0.000951939537424877 | 0.840109139938182 | NA | NA | NA | Wald ratio | TRUE |
| Citrate | Chitotriosidase-1 \|\| id:prot-a-543 | 2 | rs12411260; rs872583 | -0.00172886578341148 | 0.751371203117705 | 1.42587227527297 | NA | NA | Inverse variance weighted | TRUE |
| Citrate | Chitotriosidase-1 \|\| id:prot-a-543 | 1 | rs12411260 | 0.0263701136978885 | 0.271298368930982 | NA | NA | NA | Wald ratio | TRUE |
| Citrate | Chitotriosidase-1 \|\| id:prot-a-543 | 1 | rs872583 | -0.00278854489164087 | 0.549154329440202 | NA | NA | NA | Wald ratio | TRUE |
| Lactate | Chitotriosidase-1 \|\| id:prot-a-543 | 2 | rs12411260; rs872583 | -0.00722174908512199 | 0.116832628245503 | 0.407357193192749 | NA | NA | Inverse variance weighted | TRUE |
| Lactate | Chitotriosidase-1 \|\| id:prot-a-543 | 1 | rs12411260 | 0.0079122360584732 | 0.743242583455185 | NA | NA | NA | Wald ratio | TRUE |
| Lactate | Chitotriosidase-1 \|\| id:prot-a-543 | 1 | rs872583 | -0.00779256965944272 | 0.0966883595008454 | NA | NA | NA | Wald ratio | TRUE |
| Fasting glucose | Chitotriosidase-1 \|\| id:prot-a-543 | 2 | rs12411260; rs872583 | 0.00130654778270339 | 0.524355459291449 | 0.53255661278407 | NA | NA | Inverse variance weighted | TRUE |
| Fasting glucose | Chitotriosidase-1 \|\| id:prot-a-543 | 1 | rs12411260 | 0.00866269626421224 | 0.399729035501385 | NA | NA | NA | Wald ratio | TRUE |
| Fasting glucose | Chitotriosidase-1 \|\| id:prot-a-543 | 1 | rs872583 | 0.00100163904571116 | 0.632464543594736 | NA | NA | NA | Wald ratio | TRUE |
| Fasting insulin | Chitotriosidase-1 \|\| id:prot-a-543 | 2 | rs12411260; rs872583 | -0.0034305922958981 | 0.154222018073381 | 0.0152842264056049 | NA | NA | Inverse variance weighted | TRUE |
| Fasting insulin | Chitotriosidase-1 \|\| id:prot-a-543 | 1 | rs12411260 | -0.00487276664861938 | 0.682472947499482 | NA | NA | NA | Wald ratio | TRUE |
| Fasting insulin | Chitotriosidase-1 \|\| id:prot-a-543 | 1 | rs872583 | -0.0033691495173921 | 0.170571316384161 | NA | NA | NA | Wald ratio | TRUE |
| HDL cholesterol | Chitotriosidase-1 \|\| id:prot-a-543 | 2 | rs12411260; rs872583 | -0.00300278483743921 | 0.665872004036469 | 10.7828399000016 | NA | NA | Inverse variance weighted | TRUE |
| HDL cholesterol | Chitotriosidase-1 \|\| id:prot-a-543 | 1 | rs12411260 | -0.0387697888467786 | 0.000475869927333336 | NA | NA | NA | Wald ratio | TRUE |
| HDL cholesterol | Chitotriosidase-1 \|\| id:prot-a-543 | 1 | rs872583 | -0.00165083773447459 | 0.444133255198335 | NA | NA | NA | Wald ratio | TRUE |
| LDL cholesterol | Chitotriosidase-1 \|\| id:prot-a-543 | 2 | rs12411260; rs872583 | -5.89101136301007E-05 | 0.979700530714638 | 0.0454011776000127 | NA | NA | Inverse variance weighted | TRUE |
| LDL cholesterol | Chitotriosidase-1 \|\| id:prot-a-543 | 1 | rs12411260 | -0.00259656199242014 | 0.830535037396056 | NA | NA | NA | Wald ratio | TRUE |
| LDL cholesterol | Chitotriosidase-1 \|\| id:prot-a-543 | 1 | rs872583 | 3.69928064104899E-05 | 0.987486292499672 | NA | NA | NA | Wald ratio | TRUE |
| triglycerides | Chitotriosidase-1 \|\| id:prot-a-543 | 2 | rs12411260; rs872583 | 0.00293996860348356 | 0.711847972526887 | 12.9907863293649 | NA | NA | Inverse variance weighted | TRUE |
| triglycerides | Chitotriosidase-1 \|\| id:prot-a-543 | 1 | rs12411260 | 0.0438825663237683 | 0.000149381769574497 | NA | NA | NA | Wald ratio | TRUE |
| triglycerides | Chitotriosidase-1 \|\| id:prot-a-543 | 1 | rs872583 | 0.0013926698233473 | 0.535873815486718 | NA | NA | NA | Wald ratio | TRUE |
| Acetate | Semaphorin-3E \|\| id:prot-a-2672 | 2 | rs302121; rs3757607 | -0.00600840785591715 | 0.505962444752383 | 3.1156275748025 | NA | NA | Inverse variance weighted | FALSE |
| Acetate | Semaphorin-3E \|\| id:prot-a-2672 | 1 | rs302121 | 0.0123964603739982 | 0.285854712290867 | NA | NA | NA | Wald ratio | FALSE |
| Acetate | Semaphorin-3E \|\| id:prot-a-2672 | 1 | rs3757607 | -0.0104420742591931 | 0.0670026817557282 | NA | NA | NA | Wald ratio | FALSE |
| Citrate | Semaphorin-3E \|\| id:prot-a-2672 | 2 | rs302121; rs3757607 | -0.00513210302414077 | 0.309491591517043 | 0.0382175943640548 | NA | NA | Inverse variance weighted | FALSE |
| Citrate | Semaphorin-3E \|\| id:prot-a-2672 | 1 | rs302121 | -0.00312079252003562 | 0.785391808577453 | NA | NA | NA | Wald ratio | FALSE |
| Citrate | Semaphorin-3E \|\| id:prot-a-2672 | 1 | rs3757607 | -0.00561665476615494 | 0.318058193350081 | NA | NA | NA | Wald ratio | FALSE |
| Lactate | Semaphorin-3E \|\| id:prot-a-2672 | 2 | rs302121; rs3757607 | 0.00266690619560961 | 0.698732112475711 | 1.83346660378724 | NA | NA | Inverse variance weighted | FALSE |
| Lactate | Semaphorin-3E \|\| id:prot-a-2672 | 1 | rs302121 | -0.0113744211932324 | 0.324773475398836 | NA | NA | NA | Wald ratio | FALSE |
| Lactate | Semaphorin-3E \|\| id:prot-a-2672 | 1 | rs3757607 | 0.0060484291324527 | 0.285973048193368 | NA | NA | NA | Wald ratio | FALSE |
| Fasting glucose | Semaphorin-3E \|\| id:prot-a-2672 | 2 | rs302121; rs3757607 | 0.00375120035887386 | 0.133539234028631 | 1.13495742811834 | NA | NA | Inverse variance weighted | FALSE |
| Fasting glucose | Semaphorin-3E \|\| id:prot-a-2672 | 1 | rs302121 | 0.0091273374888691 | 0.101005166948207 | NA | NA | NA | Wald ratio | FALSE |
| Fasting glucose | Semaphorin-3E \|\| id:prot-a-2672 | 1 | rs3757607 | 0.00258836129953588 | 0.317310507862914 | NA | NA | NA | Wald ratio | FALSE |
| HDL cholesterol | Semaphorin-3E \|\| id:prot-a-2672 | 2 | rs302121; rs3757607 | 0.00283218792410247 | 0.316501187186894 | 1.44750598720229 | NA | NA | Inverse variance weighted | FALSE |
| HDL cholesterol | Semaphorin-3E \|\| id:prot-a-2672 | 1 | rs302121 | -0.00291685218165628 | 0.583857533014494 | NA | NA | NA | Wald ratio | FALSE |
| HDL cholesterol | Semaphorin-3E \|\| id:prot-a-2672 | 1 | rs3757607 | 0.00422276865405212 | 0.106875727078711 | NA | NA | NA | Wald ratio | FALSE |
| LDL cholesterol | Semaphorin-3E \|\| id:prot-a-2672 | 2 | rs302121; rs3757607 | 0.00354761095517059 | 0.304888027560719 | 1.80949797440844 | NA | NA | Inverse variance weighted | FALSE |
| LDL cholesterol | Semaphorin-3E \|\| id:prot-a-2672 | 1 | rs302121 | -0.00349038290293856 | 0.549332178345564 | NA | NA | NA | Wald ratio | FALSE |
| LDL cholesterol | Semaphorin-3E \|\| id:prot-a-2672 | 1 | rs3757607 | 0.00524633166726169 | 0.0669675990826272 | NA | NA | NA | Wald ratio | FALSE |
| triglycerides | Semaphorin-3E \|\| id:prot-a-2672 | 2 | rs302121; rs3757607 | -0.000107903807648407 | 0.964840675743304 | 0.0860191010832877 | NA | NA | Inverse variance weighted | FALSE |
| triglycerides | Semaphorin-3E \|\| id:prot-a-2672 | 1 | rs302121 | 0.00135334149599288 | 0.807389250694315 | NA | NA | NA | Wald ratio | FALSE |
| triglycerides | Semaphorin-3E \|\| id:prot-a-2672 | 1 | rs3757607 | -0.000460650660478401 | 0.86587829026934 | NA | NA | NA | Wald ratio | FALSE |
| Acetate | Semaphorin-3E \|\| id:prot-a-2672 | 2 | rs302121; rs3757607 | -0.00600840785591715 | 0.505962444752383 | 3.1156275748025 | NA | NA | Inverse variance weighted | TRUE |
| Acetate | Semaphorin-3E \|\| id:prot-a-2672 | 1 | rs302121 | 0.0123964603739982 | 0.285854712290867 | NA | NA | NA | Wald ratio | TRUE |
| Acetate | Semaphorin-3E \|\| id:prot-a-2672 | 1 | rs3757607 | -0.0104420742591931 | 0.0670026817557282 | NA | NA | NA | Wald ratio | TRUE |
| Citrate | Semaphorin-3E \|\| id:prot-a-2672 | 2 | rs302121; rs3757607 | -0.00513210302414077 | 0.309491591517043 | 0.0382175943640548 | NA | NA | Inverse variance weighted | TRUE |
| Citrate | Semaphorin-3E \|\| id:prot-a-2672 | 1 | rs302121 | -0.00312079252003562 | 0.785391808577453 | NA | NA | NA | Wald ratio | TRUE |
| Citrate | Semaphorin-3E \|\| id:prot-a-2672 | 1 | rs3757607 | -0.00561665476615494 | 0.318058193350081 | NA | NA | NA | Wald ratio | TRUE |
| Lactate | Semaphorin-3E \|\| id:prot-a-2672 | 2 | rs302121; rs3757607 | 0.00266690619560961 | 0.698732112475711 | 1.83346660378724 | NA | NA | Inverse variance weighted | TRUE |
| Lactate | Semaphorin-3E \|\| id:prot-a-2672 | 1 | rs302121 | -0.0113744211932324 | 0.324773475398836 | NA | NA | NA | Wald ratio | TRUE |
| Lactate | Semaphorin-3E \|\| id:prot-a-2672 | 1 | rs3757607 | 0.0060484291324527 | 0.285973048193368 | NA | NA | NA | Wald ratio | TRUE |
| Fasting glucose | Semaphorin-3E \|\| id:prot-a-2672 | 2 | rs302121; rs3757607 | 0.00375120035887386 | 0.133539234028631 | 1.13495742811834 | NA | NA | Inverse variance weighted | TRUE |
| Fasting glucose | Semaphorin-3E \|\| id:prot-a-2672 | 1 | rs302121 | 0.0091273374888691 | 0.101005166948207 | NA | NA | NA | Wald ratio | TRUE |
| Fasting glucose | Semaphorin-3E \|\| id:prot-a-2672 | 1 | rs3757607 | 0.00258836129953588 | 0.317310507862914 | NA | NA | NA | Wald ratio | TRUE |
| HDL cholesterol | Semaphorin-3E \|\| id:prot-a-2672 | 2 | rs302121; rs3757607 | 0.00283218792410247 | 0.316501187186894 | 1.44750598720229 | NA | NA | Inverse variance weighted | TRUE |
| HDL cholesterol | Semaphorin-3E \|\| id:prot-a-2672 | 1 | rs302121 | -0.00291685218165628 | 0.583857533014494 | NA | NA | NA | Wald ratio | TRUE |
| HDL cholesterol | Semaphorin-3E \|\| id:prot-a-2672 | 1 | rs3757607 | 0.00422276865405212 | 0.106875727078711 | NA | NA | NA | Wald ratio | TRUE |
| LDL cholesterol | Semaphorin-3E \|\| id:prot-a-2672 | 2 | rs302121; rs3757607 | 0.00354761095517059 | 0.304888027560719 | 1.80949797440844 | NA | NA | Inverse variance weighted | TRUE |
| LDL cholesterol | Semaphorin-3E \|\| id:prot-a-2672 | 1 | rs302121 | -0.00349038290293856 | 0.549332178345564 | NA | NA | NA | Wald ratio | TRUE |
| LDL cholesterol | Semaphorin-3E \|\| id:prot-a-2672 | 1 | rs3757607 | 0.00524633166726169 | 0.0669675990826272 | NA | NA | NA | Wald ratio | TRUE |
| triglycerides | Semaphorin-3E \|\| id:prot-a-2672 | 2 | rs302121; rs3757607 | -0.000107903807648407 | 0.964840675743304 | 0.0860191010832877 | NA | NA | Inverse variance weighted | TRUE |
| triglycerides | Semaphorin-3E \|\| id:prot-a-2672 | 1 | rs302121 | 0.00135334149599288 | 0.807389250694315 | NA | NA | NA | Wald ratio | TRUE |
| triglycerides | Semaphorin-3E \|\| id:prot-a-2672 | 1 | rs3757607 | -0.000460650660478401 | 0.86587829026934 | NA | NA | NA | Wald ratio | TRUE |
| Fasting insulin | C-C motif chemokine 22 \|\| id:prot-a-398 | 4 | rs1787782; rs4550435; rs77542162; rs79928291 | 0.00481425235904898 | 0.48814928672317 | 4.56600449446187 | NA | NA | Inverse variance weighted | FALSE |
| Fasting insulin | C-C motif chemokine 22 \|\| id:prot-a-398 | 4 | rs1787782; rs4550435; rs77542162; rs79928291 | -0.0110063007658161 | 0.589816479496841 | 3.04746503376428 | 0.00858772728951504 | 0.4233067427145 | MR Egger | FALSE |
| Fasting insulin | C-C motif chemokine 22 \|\| id:prot-a-398 | 4 | rs1787782; rs4550435; rs77542162; rs79928291 | -0.0017264586133199 | 0.895471530833032 | NA | NA | NA | Simple mode | FALSE |
| Fasting insulin | C-C motif chemokine 22 \|\| id:prot-a-398 | 1 | rs1787782 | -0.00236282488842216 | 0.859924253238214 | NA | NA | NA | Wald ratio | FALSE |
| Fasting insulin | C-C motif chemokine 22 \|\| id:prot-a-398 | 1 | rs4550435 | 0.0232211967847574 | 0.0511761190432773 | NA | NA | NA | Wald ratio | FALSE |
| Fasting insulin | C-C motif chemokine 22 \|\| id:prot-a-398 | 1 | rs77542162 | 0.00938176569641568 | 0.375421607696006 | NA | NA | NA | Wald ratio | FALSE |
| Fasting insulin | C-C motif chemokine 22 \|\| id:prot-a-398 | 1 | rs79928291 | -0.00823335685721007 | 0.410206998692259 | NA | NA | NA | Wald ratio | FALSE |
| Fasting insulin | C-C motif chemokine 22 \|\| id:prot-a-398 | 4 | rs1787782; rs4550435; rs77542162; rs79928291 | 0.00208307617123694 | 0.756406325048641 | NA | NA | NA | Weighted median | FALSE |
| Fasting insulin | C-C motif chemokine 22 \|\| id:prot-a-398 | 4 | rs1787782; rs4550435; rs77542162; rs79928291 | -0.00239099040265998 | 0.845966280187589 | NA | NA | NA | Weighted mode | FALSE |
| Fasting insulin | C-C motif chemokine 22 \|\| id:prot-a-398 | 3 | rs1787782; rs4550435; rs79928291 | 0.00301304911850252 | 0.757379061363478 | 4.30635562111444 | NA | NA | Inverse variance weighted | TRUE |
| Fasting insulin | C-C motif chemokine 22 \|\| id:prot-a-398 | 3 | rs1787782; rs4550435; rs79928291 | -0.025788592950994 | 0.438139238891358 | 1.37479226438713 | 0.0137517978226841 | 0.382262885462347 | MR Egger | TRUE |
| Fasting insulin | C-C motif chemokine 22 \|\| id:prot-a-398 | 3 | rs1787782; rs4550435; rs79928291 | -0.00530022925751625 | 0.695457307586205 | NA | NA | NA | Simple mode | TRUE |
| Fasting insulin | C-C motif chemokine 22 \|\| id:prot-a-398 | 1 | rs1787782 | -0.00236282488842216 | 0.859924253238214 | NA | NA | NA | Wald ratio | TRUE |
| Fasting insulin | C-C motif chemokine 22 \|\| id:prot-a-398 | 1 | rs4550435 | 0.0232211967847574 | 0.0511761190432773 | NA | NA | NA | Wald ratio | TRUE |
| Fasting insulin | C-C motif chemokine 22 \|\| id:prot-a-398 | 1 | rs79928291 | -0.00823335685721007 | 0.410206998692259 | NA | NA | NA | Wald ratio | TRUE |
| Fasting insulin | C-C motif chemokine 22 \|\| id:prot-a-398 | 3 | rs1787782; rs4550435; rs79928291 | -0.00364891244527325 | 0.672394170432556 | NA | NA | NA | Weighted median | TRUE |
| Fasting insulin | C-C motif chemokine 22 \|\| id:prot-a-398 | 3 | rs1787782; rs4550435; rs79928291 | -0.00638333183709635 | 0.604029647298022 | NA | NA | NA | Weighted mode | TRUE |
